# Dataset on economic analysis of mass production of algae in LED-based photobioreactors

**DOI:** 10.1016/j.dib.2018.12.010

**Published:** 2018-12-07

**Authors:** Weiqi Fu, Steinn Gudmundsson, Kristine Wichuk, Sirus Palsson, Bernhard O. Palsson, Kourosh Salehi-Ashtiani, Sigurður Brynjólfsson

**Affiliations:** a Center for Systems Biology and Faculty of Industrial Engineering, Mechanical Engineering and Computer Science, School of Engineering and Natural Sciences, University of Iceland, 101 Reykjavík, Iceland; b Division of Science and Math, and Center for Genomics and Systems Biology (CGSB), New York University Abu Dhabi, Abu Dhabi, UAE; c Department of Bioengineering, University of California, San Diego, La Jolla, CA 92093-0412, USA

## Abstract

The data presented in this article are related to the research article entitled “Sugar-stimulated CO_2_ sequestration by the green microalga *Chlorella vulgaris*” (Fu et al., 2019) [1]. The data describe a rational design and scale-up of LED-based photobioreactors for producing value-added algal biomass while removing waste CO_2_ from flu gases from power plants. The dataset were created from growth rate experiments for biomass production including direct biomass productivity data, PBR size and setup parameters, medium composition as well as indirect energy cost and overhead in Iceland. A complete economic analysis is formed through a cost breakdown as well as PBR scalability predictions.

## Specifications table

**Table:** 

Subject area	*Biotechnology*
More specific subject area	*Environmental biotechnology*
Type of data	*Table*
How data was acquired	*Growth rate experiments for biomass production*
Data format	*Raw, analyzed and descriptive data*
Experimental factors	*A fast-growing Chlorella strain was cultivated in LED-based PBRs and further optimized for biomass production*
Experimental features	*Experimentally derived production rate and scale-up predictions*
Data source location	*Iceland*
Data accessibility	*Data are within this article*
Related research article	“Sugar-stimulated CO_2_ sequestration by the green microalga *Chlorella vulgaris*” by Fu et al., 2019 (In press) [1].

## Value of the data

• The data show the economic feasibility of algal biomass production using artificial LED illumination.
• The economic analysis is provided to predict the limiting cost factors of algal mass production.
• The data can be used to provide a benchmark for future industrial applications of algae.

## 1 Data

To analyze and calculate the major costs of mass production of algae, it is critical to evaluate the scalability of a PBR system. The key data are from the design parameters of LED-based photobioreactor systems as well as the experimentally derived biomass productivity data (Table 1). As shown in Table 1, the costs of LED-based PBRs for producing algae are high at present and only feasible for value-added products. Detailed data are accessible through the research article [1].

**Table 1:** A summary of the major costs and related key assumptions for mass production of algae.

Production cost for one kilogram algal dry biomass	Biomass productivity (g/L/day)			PBR system a	
	Current/Expected	Achievable (Short-term)	Targeted (Long-term)	Single PBR working volume	Total volume in a plant
($)	1.4	1.82	2.8	(L)	(L)
Materials/Direct costs	12.62	9.73	6.36	22	21,780
CAPEX	10.27	7.9	5.14		
Overhead	26.27	21.36	15.63		
Total	49.16	38.99	27.13		

a The PBR system consists of 30 modules and there are 33 PBR unit for each module.

## 2 Experimental design, materials and methods

**Fig. 1:**
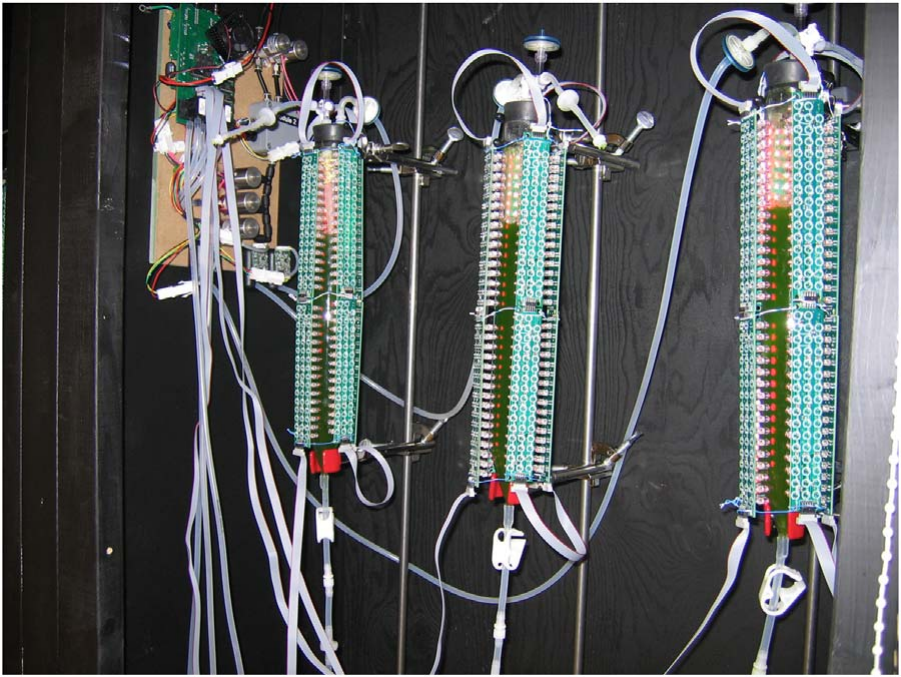
A prototype of designed PBR system highlighting its artificial LED illumination.

A bubble column LED-based PBR system was tested and used for economic analysis of mass production. The PBR systems are setup with controllable key parameters including input CO_2_ level (mixed with air), LED illumination (different spectra and intensities), temperature and pH in the medium. Artificial light supplied to the PBRs was based on LEDs consisting of narrow spectrum of red light, blue light or combined red and blue light [2]. Blue LEDs have a narrow output spectrum of 470 ± 20 or 488 ± 20 nm while red LEDs have a narrow output spectrum of 660 ± 20 or 680 ± 20 nm. The LED panels with different peaks (660 or 680 nm; 470 or 488 nm) used depend on the availability and cost in the market. A prototype of three PBR units highlighting artificial LED illumination was shown in Fig. 1. These PBR units formed the basis for growth rate experiments and showed the feature and scalability of bubble-column photobioreactors.

A fast-growing algal strain *C. vulgaris* (UTEX 26, UTEX Culture Collection of Algae) achieved in a previous study [2] was further optimized [1]. The biomass production data as well as photobioreactor design related parameter data are experimentally derived [1]. With all these direct and indirect parameters for algal mass production in LED-based PBRs including growth rate data, PBR size data, medium composition data, energy cost and overhead in Iceland, a cost breakdown is performed for a complete economic analysis.
