# Supplementary material for: Dataset on economic analysis of mass production of algae in LED-based photobioreactors
Source: Data Brief. 2018 Dec 7;22:137–9. doi: 10.1016/j.dib.2018.12.010 (PMC6299123; doi:10.1016/j.dib.2018.12.010)
Supplement: Supplementary file 1 — Supplementary material [file mmc1.docx]

November 16th, 2018

Dear Editors,

We thank you in advance for considering our revised data article entitled “A cost model for algal biomass production in LED-based photobioreactors” for publication in *Data in Brief*.

All authors: Weiqi Fu (weiqi@hi.is), Steinn Gudmundsson (steinng@hi.is), Kristine Wichuk (kwichuk@hi.is), Sirus Palsson (siruspalsson@gmail.com), Bernhard O. Palsson (palsson@ucsd.edu), Kourosh Salehi-Ashtiani (ksa3@nyu.edu) and Sigurður Brynjólfsson (sb@hi.is) have confirmed that they have no competing interests for declaration.

Sincerely,

Weiqi Fu

Principal Investigator / Adjunct Associate Professor

Center for Systems Biology

School of Engineering and Natural Sciences

University of Iceland

Sturlugata 8

101 Reykjavik, Iceland

Phone: +971-563052428

Email: [weiqi@hi.is](mailto:weiqi@hi.is)
